# The Effect of Saline Coolant on Temperature Levels during Decortication with a Midas Rex: An *in Vitro* Model Using Sheep Cervical Vertebrae

**DOI:** 10.3389/fsurg.2015.00037

**Published:** 2015-07-31

**Authors:** Asher Livingston, Tian Wang, Chris Christou, Matthew H. Pelletier, William R. Walsh

**Affiliations:** ^1^Surgical and Orthopaedic Research Laboratories, Prince of Wales Clinical School, University of New South Wales, Sydney, NSW, Australia

**Keywords:** ovine, sheep, thermal necrosis, thermal imaging, decortication, necrosis, burring, endplate preparation

## Abstract

Decortication of bone with a high-speed burr in the absence of coolant may lead to local thermal necrosis and decreased healing ability, which may negatively impact clinical outcome. Little data are available on the impact of applying a coolant during the burring process. This study aims to establish an *in vitro* model to quantitatively assess peak temperatures during endplate preparation with a high-speed burr. Six sheep cervical vertebrae were dissected and mounted. Both end plates were used to give a total of 12 sites. Two thermocouples were inserted into each vertebra, 2 mm below the end plate surface and a thermal camera set up to measure surface temperature. A 3 mm high-pneumatic speed burr (Midas Rex, Medtronic, Fort Worth, TX, USA) was used to decorticate the bone in a side to side sweeping pattern, using a matchstick burr (M-8/9MH30) with light pressure. This procedure was repeated while dripping saline onto the burr and bone. Data were compared between groups using a Student’s *t*-test. Application of coolant at the bone–burr interface during decortication resulted in a significant decrease in final temperature. Without coolant, maximum temperatures 2 mm from the surface were not sufficient to cause thermal osteonecrosis, although peak surface temperatures would cause local damage. The use of a high-speed burr provides a quick and an effective method of vertebral end plate preparation. Thermal damage to the bone can be minimized through the use of light pressure and saline coolant. This has implications for any bone preparation performed with a high-speed burr.

## Introduction

Since Hibbs’ original spinal fusion was described in 1924, most surgeons have included decortication as an integral component of the procedure (1, 2). Subsequently, decortication has been reported to unlock the osteogenic potential of cancellous bone, which can improve fusion rates (3). This can be achieved by various preparation techniques. One of the quickest and easiest methods is via the use of a high-speed burr, such as the Midas Rex. Other methods, such as manual curettage, can be difficult to perform given the limited surgical exposure in spinal procedures (4) and may be prone to incomplete decortication. Following complete manual curettage, Johnson et al. (5) have shown, in an ankle model, a histological layer of cartilage still remains. This incomplete decortication and any remaining disk tissue can decrease the rate of bony fusion (2, 6).

One concern when using a high-speed burr is the potential for thermal injury to the bone. Bone thermal necrosis has been well established histologically when bone temperature exceeds 47°C for 1 min (7, 8). Thermal osteonecrosis is a known sequela of both superficial and deep bone drilling (9) and intramedullary reaming of long bones (10).

The use of a coolant while drilling is well established (11–15), and while its use may also decrease the thermal insult from a burr during the decortication process of spinal end plates; its common use has not yet been well documented. The aim of this study is to establish an *in vitro* model capable of assessing both surface and deep temperature fluctuations and utilize it to quantitatively assess peak temperatures during endplate preparation with a burr.

## Materials and Methods

All animal tissue used for this study was acquired from animals involved in IRB approved studies not involving the cervical spine. No animals were euthanized specifically for this study. Six skeletally mature sheep cervical vertebrae were dissected and mounted in a custom made jig. Both end plates of each vertebra were used to give a total of 12 sites. Prior to testing, the vertebrae were thawed at room temperature over a 12-h period. Two K-type thermocouples were inserted into each vertebra 2 mm below and parallel to the end plate surface. The tips of the thermocouples sat adjacent to the center of the endplate.

**Figure 1:**
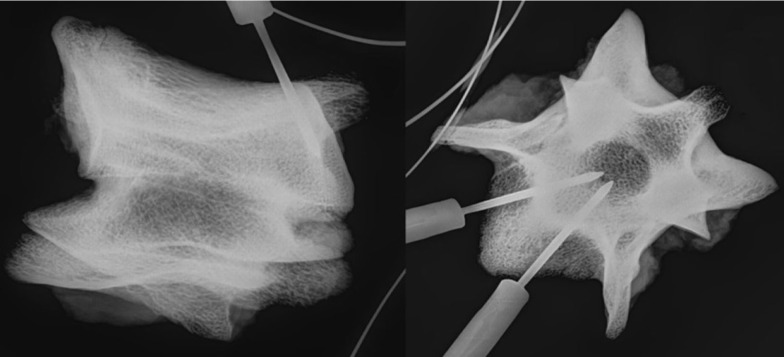
**Radiograph showing the location of the temperature probes within the vertebra in a lateral (left) and axial (right) view**.

The implantation sites were verified with high-resolution radiography (MX-20; Faxitron, Tucson, AZ, USA) (Figure 1).

**Figure 2:**
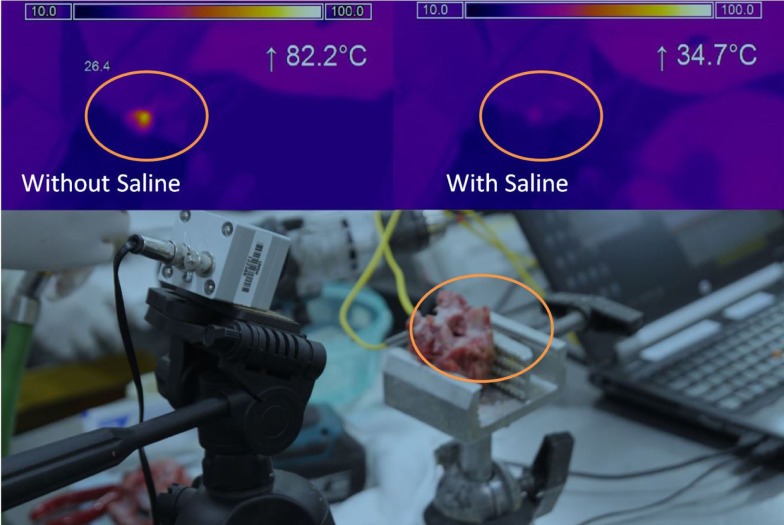
**Thermal imaging**. Camera and sample testing setup (bottom), Thermal imaging result without saline (top left), Thermal imaging result with saline (top right). Orange circle indicates the same area for the different tests.

A thermal camera (Micro-epsilon, Germany) was used to measure surface temperatures. The camera was positioned to observe the entire endplate and a region of interest (ROI) defined to encompass entire surface area (Figure 2). The temperature scale was set at 10–100°C (sensitivity of ±2°C).

A Midas Rex is a commonly available high-speed burr used in spinal surgery and was chosen to mimic surgical conditions. While downward pressure of the burr on the end plate is an additional variable, standard light pressure was used with a side to side sweeping motion to minimize this potential confounder. The decortication process was repeated with a continuous stream of saline dripping onto the burring area from a 10-ml syringe. A single operator performed all decortications. Thermocouple and thermal camera data were used to determine baseline and peak temperature. Groups were compared using a Student’s *t*-test.

## Results

The baseline temperature of the bone achieved after thawing at room temperature was 22°C at the level of the thermocouples and the surface. As expected, the application of a burr to the end plate increased temperature both at the surface of the bone and also 2 mm beneath the surface. At the level of the thermocouples, the overall temperatures reached did not approach a level high enough to achieve thermal necrosis. There was a significant difference (*p* = 0.02) in final temperature reported following burring between the coolant group (22.0 ± 1.9°C) and the group without coolant (25.0 ± 0.9°C).

**Figure 3:**
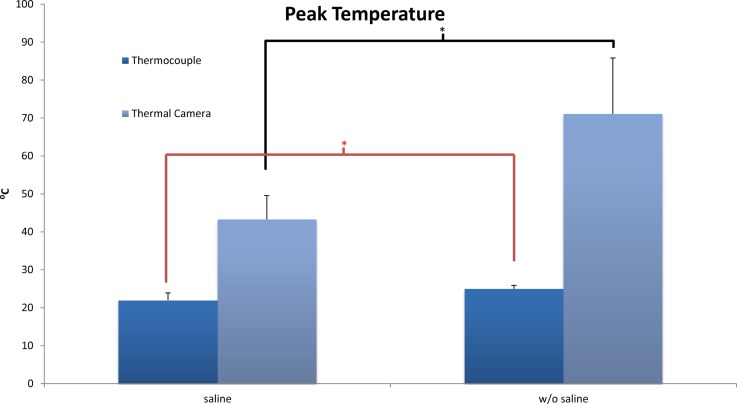
Peak temperatures as recorded by thermocouple and thermal camera systems. *p ***<*** 0.05.

Thermal camera results were analyzed to establish the change in temperature at the surface as well as peak temperature reached. With no saline coolant, mean peak temperatures of 71.1 ± 14.7°C were recorded. When coolant was applied the mean peak temperatures of 43.3 ± 6.25 were recorded. The difference between the two groups was significant (*p* < 0.01) with a maximum temperature increase of 42.0 ± 16.1°C without saline and 14.6 ± 6.1°C with saline. Unlike the thermocouple readings, peak surface temperatures did reach a level at which local thermal osteonecrosis would occur (Figure 3).

## Discussion

Joint preparation for arthrodesis and the potential for thermal osteonecrosis remain an under explored topic in current orthopedic literature. This is specifically the case with regard to end plate preparation for spinal fusion surgery. As the remaining tissues are required to heal and ultimately form a fusion, their condition is of the utmost interest and relevance.

Orthopedic research proves relevant findings for surface preparation of bone. Baker at al. (16) showed that surface reaming of femoral heads led to elevated temperatures of up to 89°C. A follow up study in 2013 was conducted, finding a potential reduction in thermal injury with external cooling and sharp instruments. Subsequently, they recommended cooling with ice-cooled water (17). They produced similar results to our model, demonstrating that with cooling, internal bone temperatures where not elevated despite high surface temperatures that could cause local thermal osteonecrosis.

Another model generated for fusion of the first metatarsal–phalangeal joint is that of sub-aquatic reaming. (18). While no adverse outcomes and an improved fusion rate were secondarily attributed to decreased thermal necrosis from a high-speed reamer, no actual temperature readings were conducted. With the current work, a specific temperature profile has now been generated for end plate preparation with a high-speed burr.

Throughout engineering literature and more recently in orthopedic and dental literature, there have been multiple studies looking at heat generation from power drills, reamers, and burrs. Several factors, such as speed, feed rate, and pressure, have been postulated as independent predictors of thermal injury (19, 20).

Increasing drill speed has commonly been attributed as a factor related to escalating thermal injury. When looking at low-speed drilling, 1800–2400 rpm, Brisman (21) found that an increase in speed or load was related to increased temperature. However, an increase in both speed and load was more efficient with no consequent increase in temperature. Kim et al. (22) established that low-speed drilling without irrigation would not produce enough heat to cause thermal necrosis. While this may be applicable to dental work, low-speed burring does not form a practical solution for vertebral end plate preparation despite its avoidance of thermal osteonecrosis.

Further increasing drill speed from 20,000 to 100,000 rpm as well as force was shown to have an inverse relationship to thermal injury (23). Thus, indicating that drilling at high speed and with large load is much more desirable than previously thought. Evidenced further, Iyer et al. (24) indicated in a comparison of low, medium, and high-speed drilling, in which high-speed drilling minimizes heat production. Natali et al. (25) also found that commercially available drill bits performed better than orthopedic drills in terms of thermal injury and subsequently surgical drills and burrs have undergone a significant design change to accommodate some of these variables over the last 15–20 years. However, a comparison of current 2 and 3 fluted drills did not show a significant difference in thermal injury despite increased drilling efficiency of the later (26).

As a reproduction of common place surgical usage, the speed of the Midas Rex (75,000 rpm) and feed rate governed by the properties of the matchstick burr, have been minimized as independent confounding variables, allowing the current study to focus on the influence of fluid cooling.

Cooling during high-speed surface preparation of bone has received little attention, while drilling has been investigated from an engineering perspective (11) and to a lesser extent a medical perspective. Drilling of bone with cooling has been shown to limit the temperature increase that can lead to osteonecrosis (12). The two common types of cooling are internal to the drill or external in the form of water or saline irrigation to the bone-burr interface.

While Augustin et al. (13) state that an internally cooled drill is ideal for orthopedics as it adequately halted the increase in bone temperature; Haider et al. (14) noted that external cooling was more effective for superficial drill holes. Superficial drilling has also been shown to generate more heat than deep drilling with external irrigation providing sufficient cooling (15).

Superficial drilling is more representative of end plate decortication; and therefore, an external cooling system as used in our model is considered most valid and is also more cost effective.

Dead or necrotic tissue cannot form part of a bony fusion without first undergoing a resorptive and remodeling process. Despite this, elevated surface temperatures are unlikely to cause a non-union in a spinal fusion. Yoshida et al. (27) showed that in bone subjected to a prolonged heat stress of 48°C, there was still new osteocyte generation albeit delayed. While the absolute temperatures generated in the present study are unlikely to cause a non-union, this may not be the case for tissues initially at body temperature.

The present study utilized two techniques to assess temperature. Both the thermal camera and the thermocouples are accurate, well established practices for the assessment of temperature. These two systems were used to assess different areas of the bone. The thermocouples were deep with the bone, which precludes the use of a thermal camera. The thermal camera assessed the surface during burring, which cannot be done directly with thermocouples. These two methods complement each other and allow the assessment of both surface and deep tissue temperature changes. The change in temperature at the surface was >2 mm within the bone, as expected. The application of heat for sort duration will cause a local peak temperature. This peak temperature will decrease the further you measure from the point of application.

This study does have some limitations. The most important to note is that the tissues were not at body temperature at the initiation of the study. Peak temperatures may have been higher if the initial temperatures had been elevated to that of the normal living tissue. Regardless of this limitation decreased peak temperatures were clearly evident when saline coolant was applied. It is important to note that warming the specimens above room temperature introduces additional variability into the model. Specimens heated above ambient temperature will exhibit a range of temperatures as they cool and may cool differently based on the size of the specimen. By allowing each sample to equilibrate with the surroundings the initial starting temperature of the sample was kept as constant as possible.

As an *in vitro* model, we do not have the potential cooling ability of normal internal blood flow. However, Wootton et al. (28) showed that during drilling, small vessel occlusion occurs rapidly, and while cortical blood flow *in vivo* may help to dissipate some heat, this is unlikely to be significant (29). Comparison between temperatures generated from *in vivo* and *in vitro* drilling has shown them to be equivalent (30), although we have not established this to be the case in this specific model.

As only light pressure was used with cooling, it would be useful to further determine if temperatures >47°C would be generated deeper in the bone with increased pressure. Coupled with an *in vivo* animal study looking at time to fusion with higher pressure end plate preparation, an applicable model for spinal surgery could be generated. Burring time ranged from approximately 20 to 30 s. During this time, the surface of the endplate experienced temperatures above 47°C for 20–50 s. With saline cooling, the time that these tissues experienced temperatures above 47°C was reduced to 0. The current study investigated saline application and temperatures during and following burring. Future studies would do well to vary burring time and pressure as well as burr type. Additionally, it would be worthwhile to investigate a minimum flow required to cap peak temperature experienced by the bone.

## Conclusion

This model has demonstrated a significant decrease in final temperature when external cooling of both the bone and burr is used during vertebral end plate decortication. Without cooling, peak surface temperatures rose to a level that is known to cause thermal osteonecrosis. This is however a local effect that was not shown at 2 mm deep to the end plates.

The use of a high-speed burr with light pressure is an efficient method for decorticating vertebral end plates in spinal fusion surgery. When used in combination with external saline coolant, it can be performed safely without increased risk of extensive thermal osteonecrosis. Further implications of this model are applicable to other areas where superficial bone preparation or decortication can be improved by the use of a high-speed burr.

## Conflict of Interest Statement

The authors declare that the research was conducted in the absence of any commercial or financial relationships that could be construed as a potential conflict of interest.

## References

[1] Ishikawa S Shin HD Bowen JR Cummings RJ. Is it necessary to decorticate segmentally instrumented spines to achieve fusion? Spine (Phila Pa 1976) (1994) 19(15):1686–90. 10.1097/00007632-199408000-00006 7973961

[2] Rihn JA Gandhi SD Sheehan P Vaccaro AR Hilibrand AS Albert TJ Disc space preparation in transforaminal lumbar interbody fusion: a comparison of minimally invasive and open approaches. Clin Orthop Relat Res (2014) 472(6):1800–5. 10.1007/s11999-014-3479-z 24522382 PMC4016455

[3] Slappey G Toribatake Y Ganey TM Ogden JA Hutton WC. Guidelines to decortication in posterolateral spine fusion. J Spinal Disord (1998) 11(2):102–9. 10.1097/00002517-199804000-00002 9588465

[4] Lin PM. Posterior lumbar interbody fusion technique: complications and pitfalls. Clin Orthop Relat Res (1985) 193:90–102. 3882302

[5] Johnson JT Schuberth JM Thornton SD Christensen JC. Joint curettage arthrodesis technique in the foot: a histological analysis. J Foot Ankle Surg (2009) 48(5):558–64. 10.1053/j.jfas.2009.05.008 19700118

[6] Li H Zou X Laursen M Egund N Lind M Bunger C. The influence of intervertebral disc tissue on anterior spinal interbody fusion: an experimental study on pigs. Eur Spine J (2002) 11(5):476–81. 10.1007/s00586-002-0455-1 12384757 PMC3611310

[7] Eriksson RA Adell R. Temperatures during drilling for the placement of implants using the osseointegration technique. J Oral Maxillofac Surg (1986) 44(1):4–7. 10.1016/0278-2391(86)90006-6 3455722

[8] Eriksson RA Albrektsson T. The effect of heat on bone regeneration: an experimental study in the rabbit using the bone growth chamber. J Oral Maxillofac Surg (1984) 42(11):705–11. 10.1016/0278-2391(84)90417-8 6593442

[9] Pandey RK Panda SS Drilling of bone: a comprehensive review. J Clin Orthop Trauma (2013) 4(1):15–30. 10.1016/j.jcot.2013.01.002 PMC3880511 26403771

[10] Henry SL Adcock RA Von Fraunhofer JA Seligson D. Heat of intramedullary reaming. South Med J (1987) 80(2):173–6. 10.1097/00007611-198702000-00008 3810211

[11] Bagci E Ozcelik B Effects of different cooling conditions on twist drill temperature. Int J Adv Manuf Technol (2007) 34(9–10):867–77. 10.1007/s00170-006-0668-2

[12] Augustin G Davila S Mihoci K Udiljak T Vedrina DS Antabak A. Thermal osteonecrosis and bone drilling parameters revisited. Arch Orthop Trauma Surg (2008) 128(1):71–7. 10.1007/s00402-007-0427-3 17762937

[13] Augustin G Davila S Udilljak T Staroveski T Brezak D Babic S. Temperature changes during cortical bone drilling with a newly designed step drill and an internally cooled drill. Int Orthop (2012) 36(7):1449–56. 10.1007/s00264-012-1491-z 22290154 PMC3385901

[14] Haider R Watzek G Plenk H. Effects of drill cooling and bone structure on IMZ implant fixation. Int J Oral Maxillofac Implants (1993) 8(1):83–91. 8468088

[15] Sener BC Dergin G Gursoy B Kelesoglu E Slih I. Effects of irrigation temperature on heat control in vitro at different drilling depths. Clin Oral Implants Res (2009) 20(3):294–8. 10.1111/j.1600-0501.2008.01643.x 19397641

[16] Baker R Whitehouse M Kilshaw M Pabbruwe M Spencer R Blom A Maximum temperatures of 89 degrees C recorded during the mechanical preparation of 35 femoral heads for resurfacing. Acta Orthop (2011) 82(6):669–73. 10.3109/17453674.2011.636681 22066558 PMC3247883

[17] Baker RP Whitehouse MR Maclean A Blom AW Bannister GC. The thermal effects of lavage on 57 ox femoral heads prepared for hip resurfacing arthroplasty. Acta Orthop (2013) 84(5):448–52. 10.3109/17453674.2013.845812 24079554 PMC3822128

[18] Moore J Berberian WS. Subaquatic reaming during arthrodesis of the first metatarsophalangeal joint to prevent thermal necrosis of bone. Orthopedics (2014) 37(6):389–91. 10.3928/01477447-20140528-04 24972427

[19] Davidson SR James DF. Drilling in bone: modeling heat generation and temperature distribution. J Biomech Eng (2003) 125(3):305–14. 10.1115/1.1535190 12929234

[20] Lee J Rabin Y Ozdoganlar OB. A new thermal model for bone drilling with applications to orthopaedic surgery. Med Eng Phys (2011) 33(10):1234–44. 10.1016/j.medengphy.2011.05.014 21803638

[21] Brisman DL. The effect of speed, pressure, and time on bone temperature during the drilling of implant sites. Int J Oral Maxillofac Implants (1996) 11(1):35–7. 8820120

[22] Kim SJ Yoo J Kim YS Shin SW. Temperature change in pig rib bone during implant site preparation by low-speed drilling. J Appl Oral Sci (2010) 18(5):522–7. 10.1590/S1678-77572010000500016 21085811 PMC4246386

[23] Abouzgia MB Symington JM. Effect of drill speed on bone temperature. Int J Oral Maxillofac Surg (1996) 25(5):394–9. 10.1016/S0901-5027(06)80040-8 8961026

[24] Iyer S Weiss C Mehta A. Effects of drill speed on heat production and the rate and quality of bone formation in dental implant osteotomies. Part I: relationship between drill speed and heat production. Int J Prosthodont (1997) 10(5):411–4. 9495159

[25] Natali C Ingle P Dowell J. Orthopaedic bone drills-can they be improved? Temperature changes near the drilling face. J Bone Joint Surg Br (1996) 78(3):357–62. 8636166

[26] Bertollo N Milne HR Ellis LP Stephens PC Gillies RM Walsh WR. A comparison of the thermal properties of 2- and 3-fluted drills and the effects on bone cell viability and screw pull-out strength in an ovine model. Clin Biomech (Bristol, Avon) (2010) 25(6):613–7. 10.1016/j.clinbiomech.2010.02.007 20359798

[27] Yoshida K Uoshima K Oda K Maeda T. Influence of heat stress to matrix on bone formation. Clin Oral Implants Res (2009) 20(8):782–90. 10.1111/j.1600-0501.2008.01654.x 19453568

[28] Wootton R Reeve J Veall N. The clinical measurement of skeletal blood flow. Clin Sci Mol Med (1976) 50(4):261–8. 1261207 10.1042/cs0500261

[29] Augustin G Zigman T Davila S Udilljak T Staroveski T Brezak D Cortical bone drilling and thermal osteonecrosis. Clin Biomech (Bristol, Avon) (2012) 27(4):313–25. 10.1016/j.clinbiomech.2011.10.010 22071428

[30] Matthews LS Hirsch C Temperatures measured in human cortical bone when drilling. J Bone Joint Surg Am (1972) 54(2):297–308. 4651263

